# Effect of different management techniques on bird taxonomic groups on rice fields in the Republic of Korea

**DOI:** 10.1038/s41598-021-01870-7

**Published:** 2021-11-16

**Authors:** Green Choi, Min Seock Do, Seok-Jun Son, Hyung-Kyu Nam

**Affiliations:** 1 MEET GREEN, Seocheon, 33646 Republic of Korea; 2 National Institute of Biological Resources, Seo-gu, Incheon, 22689 Republic of Korea; 3 Korea Institute of Wildlife Ecology, Daejeon, 34388 Republic of Korea

**Keywords:** Ecology, Environmental sciences

## Abstract

Many bird species rely on the ecological functions of rice field habitats (e.g., paddy, levee, road, and ditch). However, recent intensive practices are causing rice fields to provide fewer suitable habitats. This study examined bird habitat usage and how it is affected by cultivation methods (e.g., eco-friendly vs conventional fields). Eco-friendly and conventional rice fields in the midwestern region of the Republic of Korea were surveyed from January 2014 to December 2016, and the species presence, number of birds, and locations of observed habitats were recorded. It was found that shorebirds and herons used more eco-friendly rice paddies with lower or no pesticide and/or herbicide use, while waterfowl used paddy habitats more than the other habitats, regardless of the amounts of pesticides and/or herbicides used. Land birds used ditches or roads in conventional rice fields more than those in fields that used pesticides and/or herbicides. Pesticide and/or herbicide use affected bird taxonomic groups differently. Consequently, the use of habitats by different bird taxonomic groups varied depending on the crop cultivation as well as the cultivation methods. These results provided valuable information for managing rice fields, which serve as habitats for birds.

## Introduction

Rice fields are spatially monotonous^1,2^, yet they provide valuable habitats for several species, including aquatic plants, benthic invertebrates, and vertebrates^1,3^. They also provide essential alternative habitats for a wide range of species, including those in need of conservation, particularly due to the loss of natural habitats^4,5^. Rice production has greatly increased in recent years due to intensive practices for maximizing crop yield, but the habitat quality and biodiversity of rice fields have only decreased^6–10^. Intensive practices can be characterized by the excessive use of chemicals, such as herbicides and pesticides, expansion of farming areas, and the use of advanced machinery^11^. These intensive practices have been implemented worldwide and have severely decreased the wildlife biodiversity, such as birds, mammals, amphibians, and invertebrates^12–17^.

Rice fields can be structurally divided into paddy, levee, road, and ditch, with each component providing microhabitats with different ecological functions^6,18–20^. Of these habitats, paddies account for the largest area and can support higher species richness and abundance than the other microhabitat types^21,22^. This space can be used by various avian species depending on whether the paddy fields are flooded or dry, which varies according to the cultivation season^21,22^. The boundary between paddies is known as a levee, and it provides a necessary microhabitat that compensates for the decreased microhabitat function of the paddy when the density of rice is high during the growing season, which can prevent the access of some avian species^18,23,24^. Roads expand other microhabitats, such as levees and ditches, and form terrestrial ecosystems^6,25^. Ditches are paths that allow the irrigation of the paddy, forming aquatic ecosystems, and thus shaping the structures of the unique aquatic community, depending on the source of agricultural water, such as reservoirs and rivers^6,25^.

Pesticides and/or herbicides are intensively used in conventional rice fields, and it has been well-documented that they can directly kill avian species or indirectly reduce the abundance of food sources when used excessively^22,26–30^. Moreover, the effects of chemicals on habitat use vary among avian species^31^. In contrast, eco-friendly rice fields restrict or stop the use of pesticides and/or herbicides. Choi et al*.*^32^ reported that common greenshanks (*Tringa nebularia*) used eco-friendly paddies more than conventional paddies, while the habitat use of wood sandpipers (*Tringa glareola*) was not significantly affected by pesticide and/or herbicide use. Furthermore, some avian species such as grey herons (*Ardea cinerea*) used conventional rice fields more than eco-friendly rice fields^33^. However, previous studies have only evaluated the effects of chemicals on paddies at the species level, and no study has examined their effects on the microhabitat use (i.e., paddies, levees, ditches, and roads) of bird taxonomic groups (i.e., shorebirds, herons, waterfowl, and land birds).

The objective of this study was to evaluate the effects of chemical substance usage among intensive practices (i.e., eco-friendly vs. conventional) on different bird taxonomic groups using rice field microhabitats. We hypothesized that habitat use of shorebirds, herons, waterfowl and land birds are different according to microhabitat types (paddy, levees, ditches, and roads) in eco-friendly and conventional rice fields. In particular, we aimed to determine the (1) composition of the bird taxonomic groups using the rice fields and (2) differences in the microhabitat types used by the bird taxonomic groups in eco-friendly and conventional fields to broaden our understanding of the habitat structure and usage patterns of bird taxonomic groups in rice fields.

## Results

**Figure 1:**
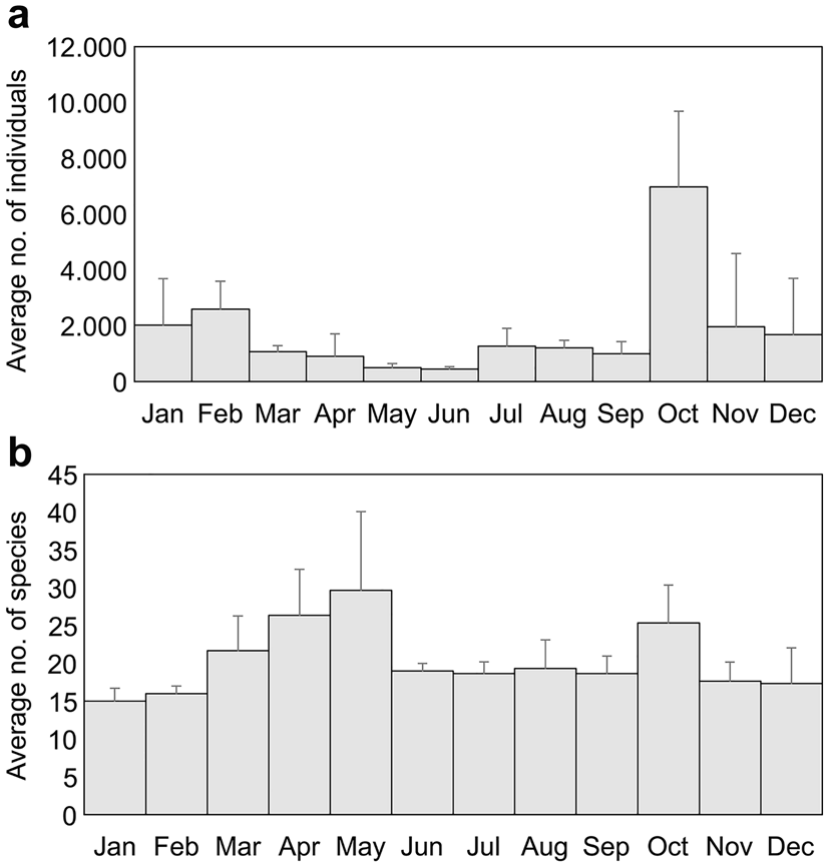
Seasonal change in the average number of (**a**) individuals and (**b**) species of birds in the rice fields of midwestern South Korea. The error bars represent the standard deviation. The sample size of each bar was 36, which is the number of surveys (once per month for 3 years, from January 2014 to December 2016).

We observed a total of 64,736 individuals of 113 species, including 22 shorebird species (822 individuals), 11 heron species (9538 individuals), 15 waterfowl species (45,036 individuals), 53 land bird species (9116 individuals), and 12 other waterbird species, including cormorants, gulls, cranes, and watercock (224 individuals) (see Supplementary Table S1). The average number of individuals was the highest in October (6973 individuals) and the lowest in June (439.3 individuals). The average number of species was the highest in May (29.7 species) and the lowest in January (15 species) (Fig. 1).

### Pattern from the self-organizing map

**Figure 2:**
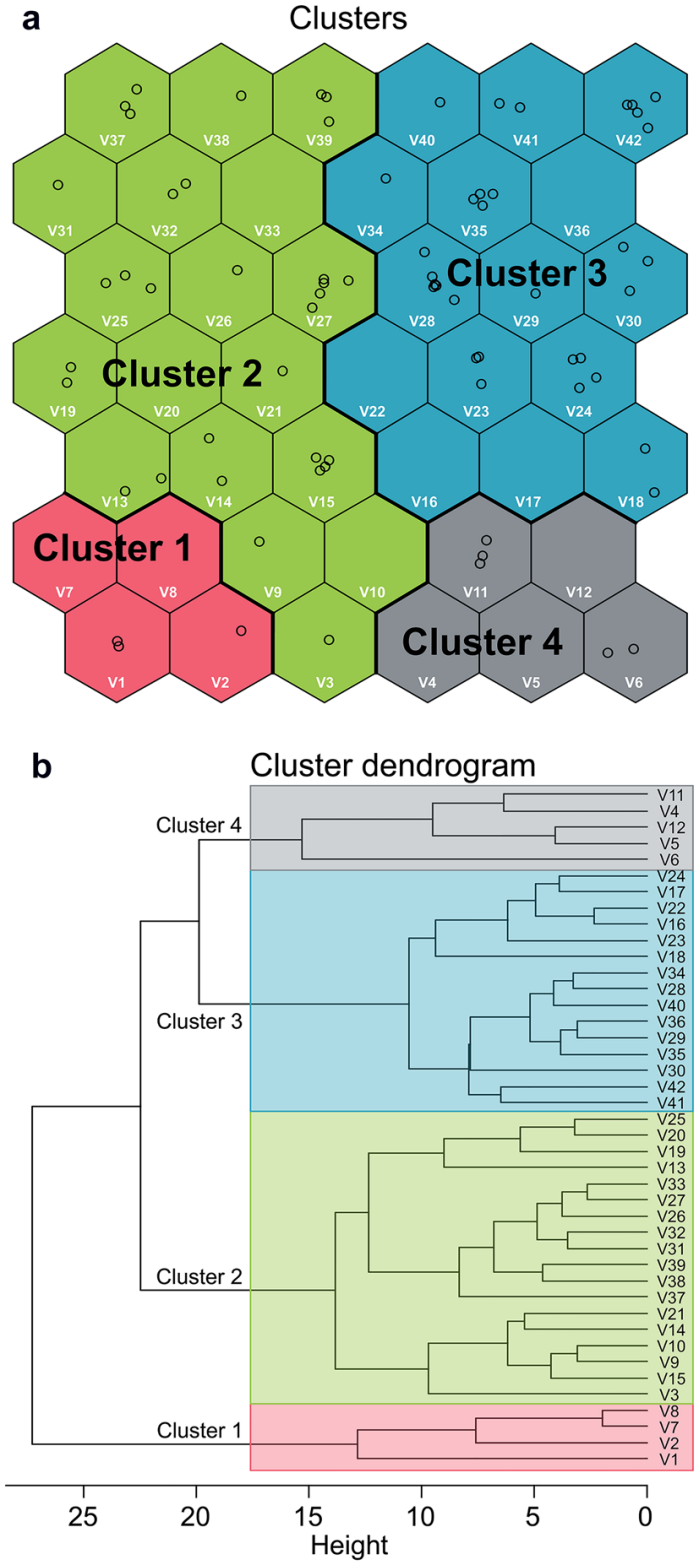
Classification of the input data (number of individuals: 113 species × 2 cultivation methods × 36 surveys) using the self-organizing map. (**a**) 42 output neurons arranged in a two-dimensional grid (7 × 6). (**b**) Neurons grouped into four clusters, which are shown in different colors, using dendrograms produced using Ward’s linkage method. V1-V42 indicate each output neuron (hexagon unit). The circles in the neurons indicate the input data, which are the bird communities observed in two areas by cultivation method during 36 surveys periods.

**Figure 3:**
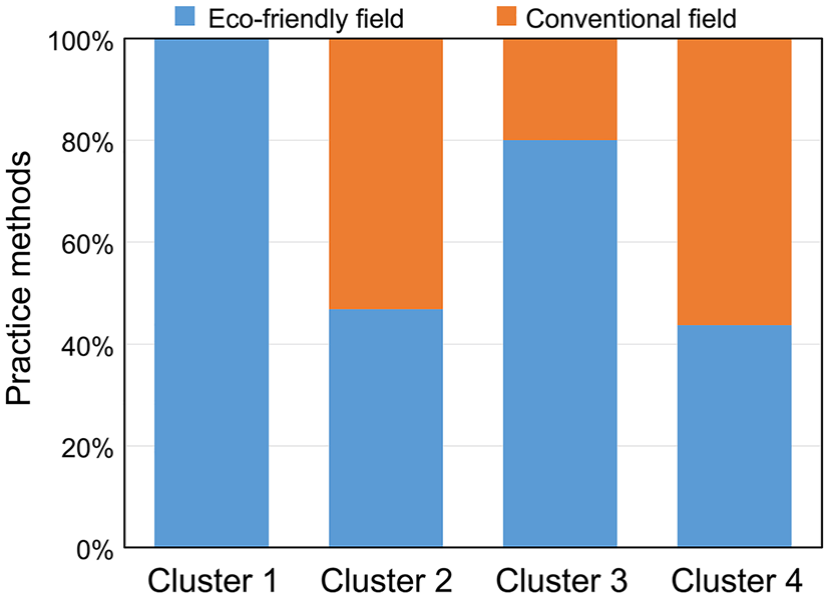
Difference in the practiced method among the four clusters defined by the self-organizing map.

**Figure 4:**
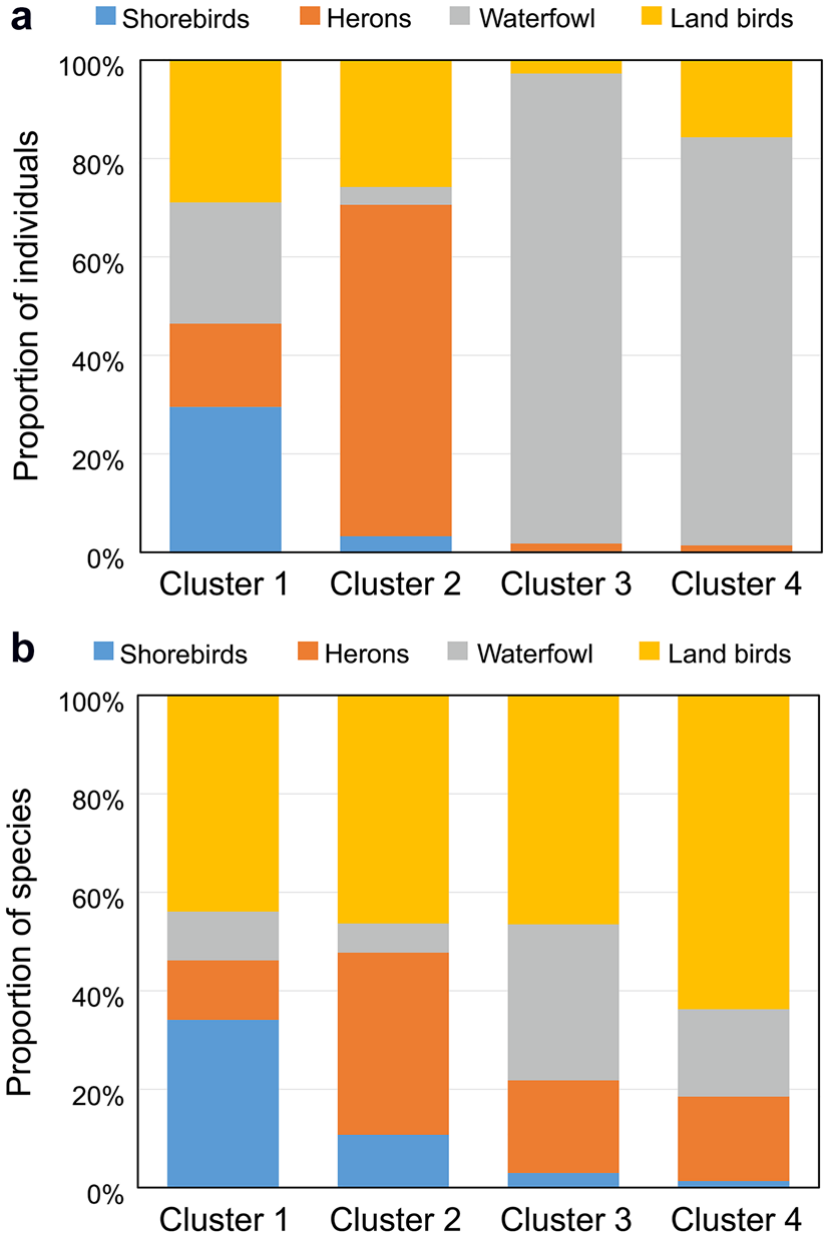
Difference in the relative numbers of (**a**) individuals and (**b**) species of bird taxonomic groups among the four clusters defined by the self-organizing map (SOM).

The 36-month survey data were divided into four clusters using a self-organizing map based on the 113 bird species observed in the eco-friendly and conventional rice fields (Fig. 2). Cluster 1 appears to include only information from eco-friendly fields, in which shorebird richness and abundance were the highest (Figs. 3 and 4). The other three clusters are formed by a combination of both field types, but with cluster 2 dominated by herons, and clusters 3 and 4 by waterfowl (Fig. 4). These clusters were significantly different in the number of individuals and species (multi-response permutation procedure (MRPP), *A* = 0.14, *P* = 0.002). Land birds accounted for the largest proportion of individuals (28.95%) and species (43.96–63.80%) in cluster 1 and all clusters, respectively (Fig. 4). Among the waterbird taxonomic groups, shorebirds (29.54% and 34.07%), herons (67.33% and 36.99%), and waterfowl (95.46% and 31.68%) accounted for the largest proportions of individuals and species, respectively, in clusters 1, 2, and 3 (Fig. 4).

### Usage characteristics of eco-friendly and conventional rice fields

**Figure 5:**
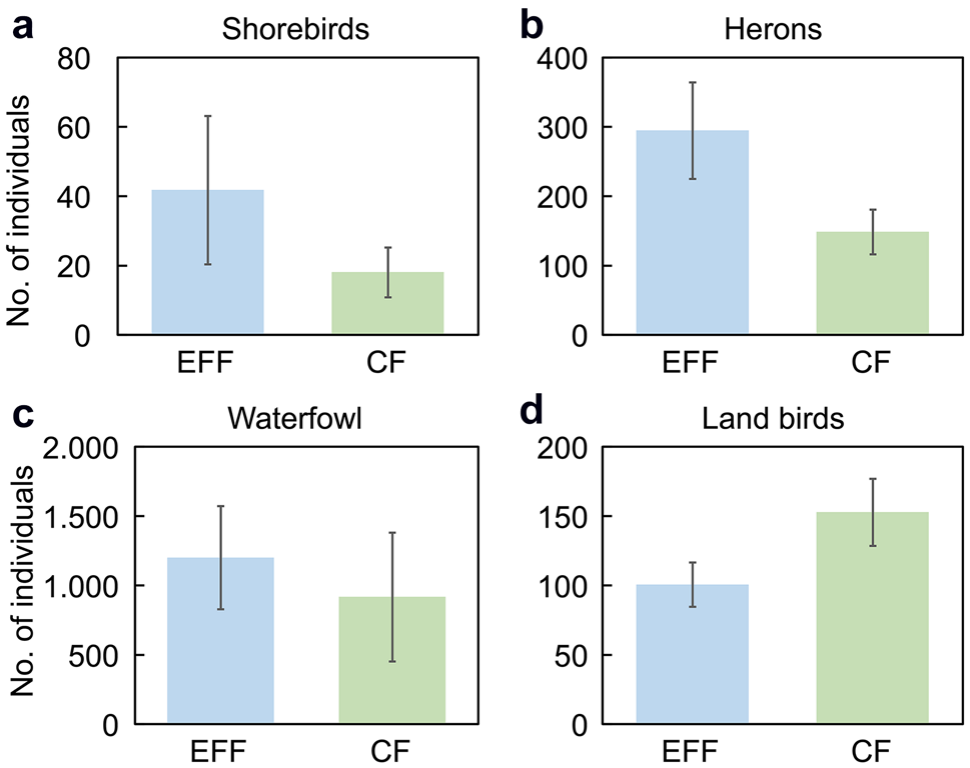
Average bird group abundance ((**a**) shorebirds, (**b**) herons, (**c**) waterfowl, and (**d**) land birds) values between eco-friendly fields (EFF) and conventional fields (CF) according to main occurrence period (shorebirds: April–May, and August–September; herons: May–October; waterfowl: October–April; land birds: all seasons). The sample size was 36, which is the number of surveys (once per month for 3 years, from January 2014 to December 2016).

**Figure 6:**
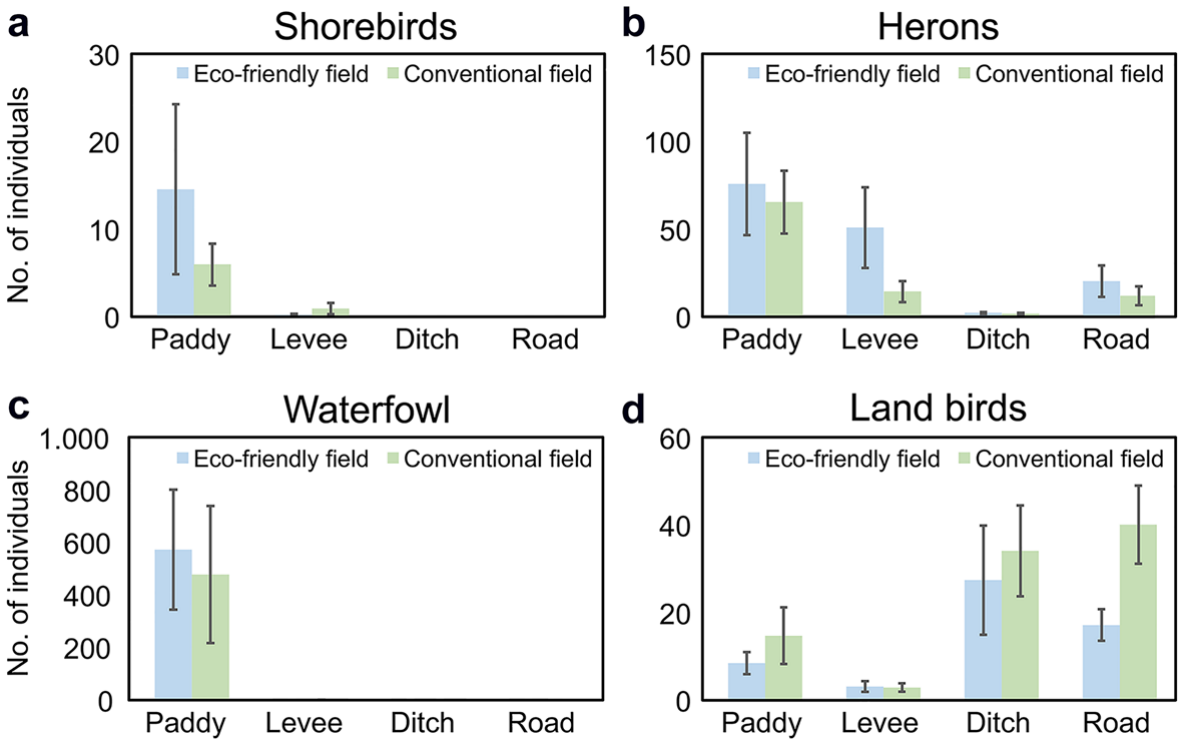
Average bird group abundance ((**a**) shorebirds, (**b**) herons, (**c**) waterfowl, and (**d**) land birds) values in eco-friendly and conventional fields among habitat types (paddy, levee, ditch, and road). The sample size was 36, which is the number of surveys (once per month for 3 years, from January 2014 to December 2016).

We found no spatial autocorrelation among the waterbirds, while land birds exhibited positive spatial autocorrelation (see Supplementary Figure S3). The generalized linear mixed model (GLMM) analysis showed that the relationship between habitat type and cultivation method varied between bird taxonomic groups (Table 1). Habitat type, cultivation method, and the interaction between habitat type and cultivation method were related to the distribution of shorebirds and herons. However, only habitat type was related to the distribution of waterfowl. The number of shorebirds and herons observed in eco-friendly rice fields was more than twice that of the observed in conventional rice fields (Fig. 5). Regardless of cultivation treatment, most shorebirds, herons, and waterfowl were observed in paddy fields, while most land birds were observed in ditches (Fig. 6). Generally, herons and waterfowl were more frequently observed in the eco-friendly than in the conventional rice fields, while more shorebirds were observed on the levees of the conventional rice field than on that of the eco-friendly fields. Land birds were observed in all microhabitats of the conventional rice fields, except levees (Fig. 6).

**Table 1:** Moran's I test for spatial autocorrelation and results of the generalized linear mixed models used to examine the impacts of cultivation method and habitat type on the abundance of bird taxonomic groups in rice fields.

Bird taxonomic groups	Moran's I test				Generalized linear mixed models					
	Observed	Expected	SD	*P*-value	Variables	Estimate	SE	χ^2^	*df*	*P*-value
Shorebirds	− 0.020	− 0.008	0.017	0.490	Habitat	1.126	0.134	0.692	3	** < 0.001**
					Practice methods	1.113	0.302	4.484	1	**0.032**
					Habitat × Practicemethods	1.613	0.290	4.497	3	**0.034**
Herons	0.002	− 0.001	0.003	0.170	Habitat	0.037	0.147	125.970	3	** < 0.001**
					Practice methods	0.451	0.125	95.798	1	** < 0.001**
					Habitat × Practice methods	1.136	0.128	172.630	3	** < 0.001**
Waterfowl	0.006	− 0.003	0.007	0.190	Habitat	0.357	0.518	36.529	3	** < 0.001**
					Practice methods	2.401	0.398	2.760	1	0.10
					Habitat × Practice methods	2.511	1.648	2.202	3	0.33
Land birds	0.012	− 0.003	0.006	**0.010**	Habitat	1.604	0.160	189.210	3	** < 0.001**
					Practice methods	1.073	0.180	0.112	1	0.74
					Habitat × Practice methods	1.554	0.175	28.333	3	** < 0.001**

For the analyses of the bird taxonomic groups, the response variable is the number of individuals in each field. The explanatory variables are the habitat type (paddy, levee, ditch, and road) and practice method (eco-friendly and conventional), and the random variables are the survey period and field location. The bold fonts represent statistical significance.

## Discussion

We identified differences in rice field use by bird taxonomic groups. The bird taxonomic groups had clearly different usage patterns and were differently affected by habitat type and cultivation method. Specifically, the microhabitat types (paddy, levee, ditch, and road) and management practice (eco-friendly and conventional field) provided unique habitat qualities that are used differently by bird taxonomic groups according to their habitat characteristics^6,18–20^. All bird taxonomic groups identified in this study used different microhabitat types, and each habitat fulfilled different functions for waterbirds and land birds. Furthermore, even for the same microhabitat type, the level of habitat use depended on the management practice.

Waterbirds, such as shorebirds, herons, and waterfowl, mostly used paddies, while land birds more frequently used ditches or roads. Paddies, which were heavily used by waterbirds, account for the largest area of rice fields and they change abruptly during the rice cultivation process^21,22,24^. This process affects the aquatic and terrestrial ecosystems, alternating them in the paddy fields^21,22,24^. Various food sources for waterbirds such as benthic organisms and spilled grains are repeatedly and periodically available, maintaining a frequent paddy use by waterbirds^34–36^.

Land birds used roads and ditches more than paddy fields. The narrow edge areas of roads and ditches have well-developed, diverse herbaceous plants, such as farm crops, reeds, and silver grass, which can be used for foraging or as resting spaces^18,19^, while the paddy is a space for growing rice, a single crop, and has a homogeneous vegetation structure^18,19^. It has been reported that higher biodiversity of herbaceous species, including crops, attracts more diverse taxa^3^, such as bird-feeding spiders, butterflies, and surface-roaming insects^37,38^. Therefore, land birds tend to use roads and ditches that offer a more diverse habitat^3^. In September, land birds were more abundant in the conventional fields than in the eco-friendly fields when the crops in the study areas matured (maximum counts, eco-friendly field = 97, conventional filed = 773; see Supplementary Table S2).

Eco-friendly cultivation paddies, which are used frequently by shorebirds, have a higher diversity of benthic organisms, a potential food source, than conventional cultivation paddies^39,40^. Even though these paddies had similar shapes as those of conventional fields, shorebirds used them considerably more because of the larger amount of available food. In contrast, shorebirds used levees in conventional cultivation fields more than levees in eco-friendly cultivation fields, which could be related to accessibility. In the area studied, the levees were managed in different ways according to the cultivation method. In eco-friendly fields, weeds were either left untouched or removed using a weeder, i.e., without complete removal, whereas herbicides were used in conventional fields, allowing for complete removal of weeds^43^. The levees in the conventional fields presented an open shape, in which shorebirds were more abundant, probably because they have a habit of foraging or resting in open environments^41,42^. Herons used the paddies, levees, and roads of the eco-friendly fields more than those of the conventional fields, because eco-friendly fields provided a higher abundance of potential food sources than the conventional fields^39,44^. Moreover, herons rarely used the ditch microhabitat because many of the ditches were concrete-lined, resulting in reduced food availability, which made them less attractive to herons than other microhabitats^6,18,19^. Waterfowl also used microhabitats of eco-friendly fields more than those of conventional fields, which could be because some paddies in the eco-friendly fields were watered, even after harvesting. Watered paddies are an important feeding ground for various waterfowl in winter^45–47^, where they were observed to be concentrated. Although land birds used all habitat types, they used conventional fields more than eco-friendly fields, a trend contrary to waterbirds. Certain farm crops grown in the conventionally cultivated rice fields at the study site (cultivated using pesticides) were not grown in the eco-friendly fields. These farm crops provided land birds with more diverse habitats in the conventional fields than in the eco-friendly fields and allowed them to forage or rest more^38^.

It has previously been found that rice cultivation techniques, such as pesticide and/or herbicide use and crop cultivation around paddies, could affect birds using rice fields^22,27,41,45,48,49^. The results of this study also support these findings, as it was found that waterbirds were more abundant in eco-friendly fields due to the larger amount of potential food^21,30,33^. It was also found that land birds were more abundant in fields that cultivated a higher number of plant crop species, which provided more diverse habitats, even if pesticides and/or herbicides were used. This increased habitat diversity was possible due to use of pesticides, which allowed various other farm crops to be grown. However, it does not mean that pesticide use is important. The various habitat structures created by the crops are important, and it would be better to have a variety of habitat structures without the use of pesticides.

In conclusion, this study reported that bird taxonomic groups in rice fields differ based on the agricultural techniques used, as they affect the habitat characteristics. As natural wetlands disappear, paddies provide alternative habitats for various birds. Rice paddies are artificial wetlands that can be changed more drastically through active human management over a short period of time than natural wetlands. Therefore, it is very important to understand the ecological characteristics and structure of paddy wetlands. The results of this study will be valuable for establishing management plans for several bird taxonomic groups that use rice fields. We concluded that (1) a combination of habitats and practice methods is considered for shorebirds and herons, (2) paddies are more important than any other habitat (e.g., levee, ditch, and road) for shorebirds and waterfowl, (3) herons use more diverse habitats than other waterbirds, (4) habitats and practice methods should be simultaneously considered for land birds, and (5) farm crops on the narrow edge areas of roads are important in rice fields. Consequently, seeking and implementing clear and systematic management plans for rice field habitats can contribute to the immediate protection and conservation of bird taxonomic groups.

The bird taxonomic groups are affected by a variety of factors such as soil management, water management, distance from breeding colonies and landscape structures between eco-friendly and conventional rice fields^17,50^. Therefore, future studies should focus on investigating the effects the management practices considering the other relevant factors. Moreover, further studies are also needed to confirm whether the effects of cultivation methods at the community level would also be valid at the species level.

## Materials and methods

### Study sites

This study was conducted in rice fields located in Nanjido-ri, Seokmun-myeon, Dangjin-gun, Chungcheongnam-do (37°02ʹN, 126°30ʹE: midwestern region of the Republic of Korea; Supplementary Figure S1). The rice fields were reclaimed in 1979, and developed by the Large-scale Comprehensive Agricultural Development Project, with a total area of 3904 ha. Rice was initially cultivated using conventional methods in the entire areas, but since 1999, eco-friendly cultivation methods have been used in some areas (574.2 ha). The eco-friendly and conventional rice fields are adjacent to one another, separated by roads, and irrigated by the same ditch^51^. The survey unit was the standardized field unit enclosed by levees and farm roads, measuring 0.45 ha ± 0.07 ha (mean ± SE). For this study, 446 eco-friendly rice fields and 442 conventional rice fields were selected.

The microhabitat types of rice fields were divided into paddies, levees, roads, and ditches. Cultivation activities (e.g., plowing, harrowing, and rice planting) are conducted throughout the season in this area, and some eco-friendly rice paddies are watered even after rice harvesting. Levees and roads are managed differently between eco-friendly and conventional rice fields. Generally, eco-friendly management includes both low-pesticide and organic farming; high pesticide use is defined as a feature of conventional farm management. Weeds on the levee and road of the eco-friendly rice fields were either left or physically removed using a weeder, while those on the conventional rice fields were removed using a chemical herbicide. In rice fields, roads were cultivated with farm crops along the narrow edge areas. Seventeen types of farm crops (e.g., soybeans, sesame, corn, leeks, perilla, napa cabbage, and spinach) were cultivated on conventional roads (Supplementary Figure S2). Farm crops were not grown in the eco-friendly levees or roads, even though some rice fields used pesticides. The period of the harvest of farm crops was mainly from September to October (Supplementary Table S2). The ditches consisted of concrete, but some, such as drainage ditches, consisted of soil in both the eco-friendly and conventional rice fields. Reeds (*Phragmites australis*) or silver grasses (*Miscanthus sinensis*) were grown in ditches composed of soil. Although some eco-friendly rice fields used pesticides (tiadinil), most did not (approximately 20 fields). Conventional rice fields used pesticides containing tiadinil, clothianidin, pyrazosulfuron-ethyl, or fentrazmide in all areas (Supplementary Table S3).

### Bird and habitat survey

Birds were surveyed once per month for 3 years from January 2014 to December 2016. The survey was conducted between 06:00 and 12:00 when visiting eco-friendly (446 fields) and conventional rice fields (442 fields), or along the road; all birds observed on the left and right sides were recorded, along with the field type they were found in. To minimize the misidentification of birds, the observation area was limited to one field (approximately 100 m in length) adjacent to the road. A total of 888 fields were observed per month.

Birds that were flying or sitting on utility poles or wires were excluded from the record, but raptors and swallows (*Hirundo rustica*) were recorded, even if they were flying. The recorded birds were divided into waterbirds and land birds based on field observations and habitat use obtained from previous studies^18^. Waterbirds were further subdivided into shorebirds, herons, and waterfowl. The habitat type of the flying swallows was recorded as paddy. For raptors, the habitat used for food acquisition or resting was recorded as the habitat type. To minimize bias due to weather, the survey was not conducted on rainy or snowy days.

### Statistical analysis

This study used a self-organizing map, which is an artificial neural network using unsupervised learning, to identify the characteristics of bird taxonomic groups observed for 3 years from 2014 to 2016^52^. Self-organizing maps are effective for exploratory data analysis^53^. In particular, they are widely used to understand the distribution or structure of animal assemblages and are suitable for data classification, patternization, and visualization^54^. They are robust, even in the presence of outliers, and their results are easy to understand and interpret^55^. Self-organizing maps are computed through an adaptive learning process that removes noise from the dataset^56^. They are connected in the direction from the input layer to the output layer, and the results of neurons in the output layer have a connection strength that is updated by unsupervised learning. The neurons in the output layer have “winner-takes-all” structures, and neurons with higher similarity to the connection strength are arranged closer to the output neurons on a two-dimensional hexagonal grid^57^.

The data (number of individuals: 113 species × 2 cultivation methods × 36 surveys) were assigned to the input layer of the self-organizing map after log-transformation (log(1 + number of individuals)) due to the differences in the number of individuals of each species. The number of output layer neurons was determined by the heuristic rule of 5 $\sqrt{n}$, as suggested by Vesanto et al*.*^58^, where n is the number of data points in the input layer (72 surveys). The final model generated 42 output neurons (six horizontal and seven vertical). After the self-learning of the self-organizing map, neurons were classified into groups based on the similarity of each neuron. The distance between neurons was measured by the Euclidean distance, and groups were classified using the Ward linkage method^54^. An MRPP was used to evaluate significant differences between groups.

A generalized linear mixed model (GLMM, Poisson distribution, and log link) was used to evaluate the effects of microhabitat type (i.e., paddy, levee, ditch, and road) and cultivation method (eco-friendly and conventional) on the number of birds per taxonomic group. Spatial autocorrelation analysis was conducted to examine the spatial characteristics of the data before applying the GLMM at a fine spatial scale (field scale). We tested the spatial autocorrelation using Moran’s I statistics^59^. When the result of the spatial autocorrelation analysis was significant, GLMM analysis was conducted by applying spatial autocorrelation. In the GLMM, the number of birds per taxonomic group was designated as a response variable, and the habitat type and cultivation method were considered as fixed effects. The timing of the survey and the location of each field were treated as random effects.

All analyses were performed using R statistical software V 3.6.1^60^. The “kohonen”^61^, “vegan”^62^, “lme4”^63^, and “spaMM” packages^64^ were used for the self-organizing map analysis, MRPP, GLMM without autocorrelation, and GLMM with autocorrelation, respectively.

## Supplementary Information

Supplementary Information.

## Data Availability

All collected data are provided in the supplementary information or available upon request directed to corresponding authors.
