# Supplementary material for: Effect of different management techniques on bird taxonomic groups on rice fields in the Republic of Korea
Source: Sci Rep. 2021 Nov 16;11:22347. doi: 10.1038/s41598-021-01870-7 (PMC8595725; doi:10.1038/s41598-021-01870-7)

**Effect of different management techniques on bird taxonomic groups on rice fields in the Republic of Korea**

**Short Title**: Bird taxonomic groups in rice fields

Green Choi^1^, Min Seock Do^2^, Seok-Jun Son^3^, Hyung-Kyu Nam^2*^

^1^ MEET GREEN, Seocheon 33646, Republic of Korea

^2^ National Institute of Biological Resources, Seo-gu Incheon 22689, Republic of Korea

^3^ Korea Institute of Wildlife Ecology, Daejeon 34388, Republic of Korea

*Corresponding author:

Hyung-Kyu Nam

Phone: +82-32-590-7239

Fax: +82-32-590-3867

E-mail: [namhk2703@korea.kr](mailto:namhk2703@korea.kr)

Supplementary Table S1. Comparison of the number of species and individuals observed in the eco-friendly and conventional rice fields in the study area.

|  | Eco-friendly rice fields | | Conventional rice fields | |
| --- | --- | --- | --- | --- |
|  | No. of species | No. of individuals | No. of species | No. of individuals |
| All birds | 104 | 36,347 | 71 | 28,389 |
| Shorebirds | 21 | 525 | 14 | 297 |
| Herons | 11 | 6,313 | 8 | 3,225 |
| Waterfowl | 14 | 25,791 | 7 | 19,245 |
| Land birds | 47 | 3,621 | 37 | 5,495 |
| Others | 11 | 97 | 5 | 127 |

Supplementary Table S2. Sowing and harvest periods of each cultivated crop in the study area.

| Crop | Sowing | Harvest |
| --- | --- | --- |
| Soybean | Late April | October |
| Sesame | May | Late August – early September |
| Red pepper | Mid May | October |
| Corn | Late April | Early October |
| Lettuce | Early May | October |
| Radish | Mid-March | November |
| Sweet potato | Mid May | September |
| Leek | Mid-March | September |
| Pumpkin | Mid May | August – September |
| Perilla | Mid-April | July – September |
| Sorghum | Early May | September – mid-October |
| Mugwort | Mid-August | September – October |
| Spinach | Late July | September – October |
| Bellflower | May | June – July |
| Eggplant | Mid May | July – October |
| Green onion | Late March | September – early November |
| Cabbage | Late April | Early August |

Supplementary Table S3. Comparison of the characteristics of eco-friendly and conventional rice fields in the study area.

|  | Eco-friendly rice field | Conventional rice field |
| --- | --- | --- |
| Rice cultivation method | Immediately adjacent and have identical cultivation stages and timing | |
| Pesticide use | Used in very limited lots | Herbicides and pesticides used in all lots |
| Pesticide ingredient | Tiadinil | Tiadinil, clothianidin, pryazosulfuron-ethyl, fentrazmide |
| Cultivated farm crops | None | Soybean, sesame, red pepper, corn, lettuce, radish, sweet potato, leek, pumpkin, perilla, sorghum, mugwort, spinach, bellflower, eggplant, green onion, and cabbage |
| After harvesting | Watered | Dry paddy |
| Road | Concrete or soil | Concrete or soil |
| Levee | Physical removal using a weeder or no removal effort | Chemical removal using herbicide |

Supplementary Table S4. R code for fitting the generalized mixed linear model (GLMM).

library(spaMM)

# fit the model

m_spamm <- fitme(Abundance ~ Practicemethods + Habitat+ Matern(1 | x + y), data = data, family = "poisson")

# x and y is position coordinates

Supplementary Figure S1. Map of the study sites (Adobe Illustrator 2021, https://www.adobe.com/)

Supplementary Figure S2. Images of the crops cultivated in rice fields. (a) Corn, (b) green onion, (c) leek, (d) cabbage, (e) soybean, and (f) red pepper.


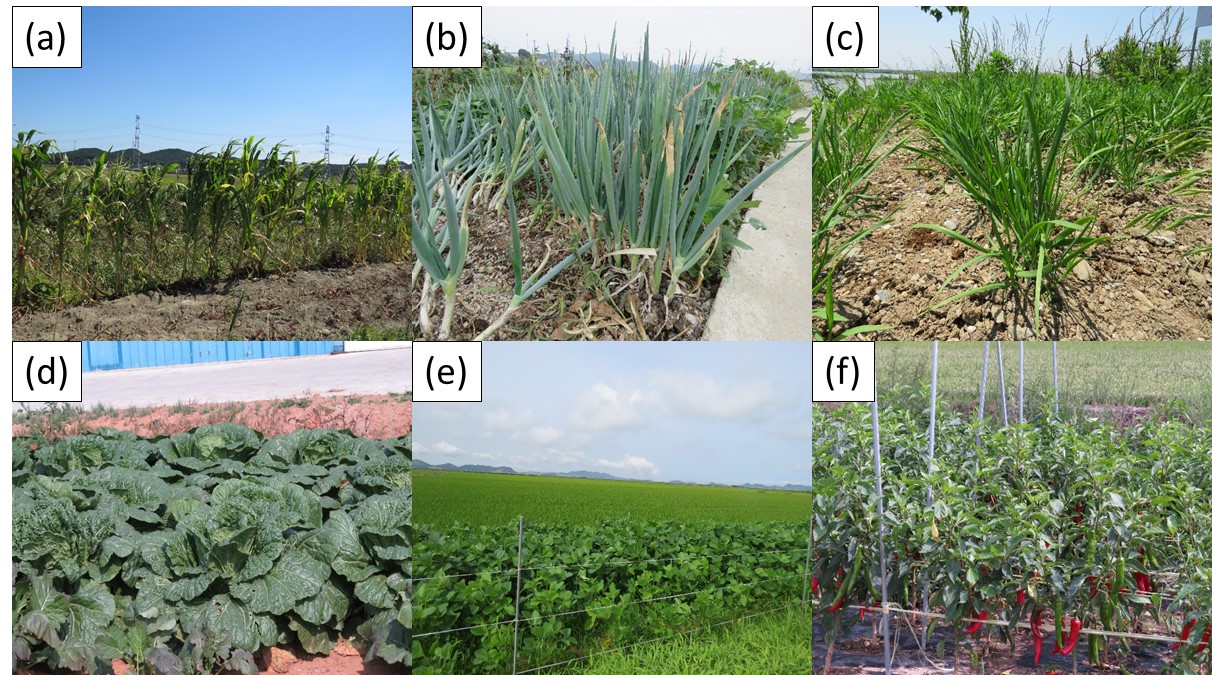


Supplementary Figure S3. Bubble plot of the spatial distribution of the full model’s residuals in land birds using rice fields.


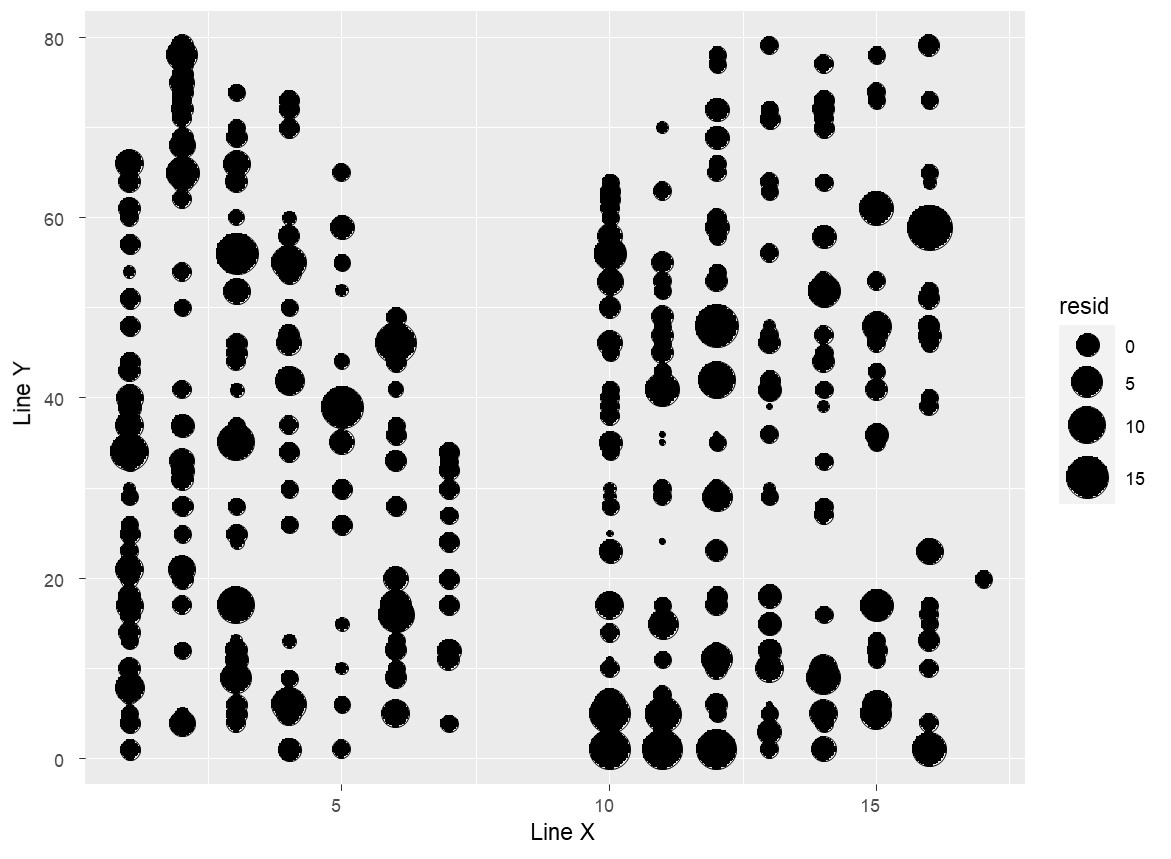

Supplement: Supplementary file 1 — Supplementary Information. [file 41598_2021_1870_MOESM1_ESM.docx]
